# The Legacy of Logging—Estimating Arboreal Lichen Occurrence in a Boreal Multiple-Use Landscape on a Two Century Scale

**DOI:** 10.1371/journal.pone.0028779

**Published:** 2011-12-16

**Authors:** Tim Horstkotte, Jon Moen, Tomas Lämås, Timo Helle

**Affiliations:** 1 Department for Ecology and Environmental Science, Umeå University, Umeå, Sweden; 2 Department of Forest Resource Management, Swedish University of Agricultural Sciences, Umeå, Sweden; 3 Finnish Forest Research Institute, Rovaniemi, Finland; University of Hull, United Kingdom

## Abstract

In northern Sweden, the availability of arboreal lichens (*Bryoria fuscescens, Alectoria sarmentosa*) as winter grazing resources is an important element in reindeer husbandry. With the industrialization of forestry, forests rich in arboreal lichens have diminished considerably. Here, we analyze how forestry has impacted lichen availability from the 1920's to the present day and model its future development assuming different forest management scenarios.

We recorded the current occurrence of *B. fuscescens* in 144 sampling plots, stratified by forest age class and dominant tree species in a 26,600 ha boreal forest landscape that is used for both reindeer herding and forestry. Lichen abundance was visually estimated in four classes: none, sparse, moderate and abundant. A binary logistic model using forest age as the independent variable was developed to predict the probability of lichens being present. Using this model, we found that lichens were present in stands that are at least 63 years old. Because of the relative paucity of stands rich in arboreal lichens, it was not possible to reliably determine how age affects the variation in abundance of older forest stands. The historical development of forests where arboreal lichens could potentially occur was studied using historic forestry records dating back 80 years. Between 1926 and the present day, forestry has reduced the cover of forests older than 60 years from 84% to 34%. The likely future spatial coverage of these stands over the next 120 years was estimated for two different management scenarios and an unmanaged reference scenario, using the Heureka strategic planning program. Under both the “business as usual” scenario and that involving more intensive forestry, continued decreases in lichen availability are projected. Our results emphasize the importance of alternative forestry practices, such as prolonged rotation periods, to increase the availability of arboreal lichens as a grazing resource for reindeer.

## Introduction

Many natural resources are simultaneously exploited by several actors, including indigenous peoples and industry, sometimes with ensuing conflicts. Such multiple-use situations require collaborative management strategies involving all of the affected stakeholders if the focal resources are to be shared sustainably and in a way that minimizes adverse environmental impact [1], known as co-management. Where indigenous peoples are involved, their political autonomy is seen as required for their cultural identity and effective contribution resource management [2]. While indigenous peoples often account for a minority of the total population, Lane [3] argues that in western post-settler states, in which the descendants of former colonizers now form the “nation”, the primary challenge of resource sharing is less a problem of approving indigenous rights and self-determination. It is rather the reasonable and realistic way of allocating resources between different claimants in planning and administrative processes. The contrasting demands placed upon natural resources, as perceived by indigenous peoples and other extractors necessitate a trade-off between commercial uses of a landscape and other values, such as social, cultural and biological values [4].

This is the situation in the boreal forests of Northern Sweden, where reindeer husbandry (*Rangifer t. tarandus* L.) is practiced by the indigenous Sami people. Throughout northern Fennoscandia, reindeer husbandry is of high cultural relevance to the Sami. In Sweden, this form of pastoralism, migrating between summer grazing grounds in the western Scandic mountains and winter grazing in boreal forests near the Baltic coast, became established in the 17^th^ century, albeit in a different form than practiced today [5]. Ever since, reindeer husbandry has undergone numerous changes and adaptations [6]-[8]. During the last century in particular, its demands have increasingly come into conflict with those of other forms of land use. For example, the constructions of hydroelectric power plants and dams submerged valuable pasture lands, calving grounds and traditional migration routes between summer and winter pastures [9]. Moreover, clear-cutting of forests was introduced on a large scale in northern Sweden during the 1950's and was subsequently made mandatory by the Forestry Act of 1979 [8] to replace natural fire-driven disturbance dynamics. However, modern clear-cutting is far more extensive than any natural disturbance pattern [10]. Selective logging of forests, which used to be relatively common, results in slower tree regeneration than does clear-cutting with subsequent soil preparation, for instance by soil scarification. As a consequence, managed forests have become much denser, while the mean age of the trees within them has decreased considerably [11]. Although these changes increase the profitability of timber production, this industrialized forestry has had certain adverse ecological consequences and has eliminated certain niches [12]. Importantly for reindeer husbandry, this silvicultural intensification has decreased the abundance of the most important winter forage for reindeer, i.e. terrestrial and arboreal lichens [13], [14], and degraded the carrying capacity of winter grazing grounds [15].

In winter, snow cover affects the availability of forage [13], [16] and the energy expended by the deer while digging for lichens beneath the snow [17]. Under certain circumstances, pastures can become inaccessibly to reindeer due to the formation of thick ice crusts. This is particularly common during late winter and early spring, after prolonged maturation of the snow cover or after weather events such as freeze-thaw-cycles [18] or rain-on-snow [19]. The availability of arboreal lichens such as *Bryoria fuscescens* and *Alectoria sarmentosa* is essential to the mitigation of such critical situations, during which the wind throw of lichen-bearing trees and the litter fall of arboreal lichen fragments becomes an important source of reindeer forage [20], [21]. Arboreal lichens are more abundant in old-growth forests [22], [23], which have become significantly less widespread in Northern Sweden as a result of specific forest management practices [24], [22].

Decreases in the abundance of arboreal lichens can increase the need for supplementary feeding and/or necessitate a reduction in herd size [25], especially during winters with adverse snow conditions. As reported by Helle & Jaakkola [7], abundant arboreal lichens have historically sustained reindeer herds throughout the winter, even under highly adverse snow conditions. Switching to arboreal lichens therefore was an adaptive strategy that allowed reindeer to overcome shortages of ground lichens. Consequently, the capacity of reindeer husbandry to adapt to adverse snow conditions increases with the ecological variety of the available pastures [26]. The transition to a highly fragmented and, on the stand level, less diverse landscape has negatively affected the abundance of arboreal lichens, primarily due to their slow growth, low dispersal rates, and strong dependence on the availability of suitable growing substrates [27].

Today, the reduced availability of this resource has strong implications for the sustainability of reindeer husbandry [28], [7], i.e. the ability to maintain its productivity in the face of disturbance events [29]. Here, we investigate how the transformation of the boreal landscape over the last century (1926–2006) by commercial forestry has affected arboreal lichens in a reindeer herding area, and simulate possible future conditions. We do this by analyzing how the potential habitat of arboreal lichens has changed over time by (i) identifying relationship between forest age and arboreal lichen abundance, and by (ii) analyzing the change in these habitat factors due to forest management over the last 80 years. Finally (iii), we simulate the future development of the potential arboreal lichen habitat over the next 120 years under various different scenarios with differing intensities of forestry.

## Materials and Methods

### Study site

**Figure 1:**
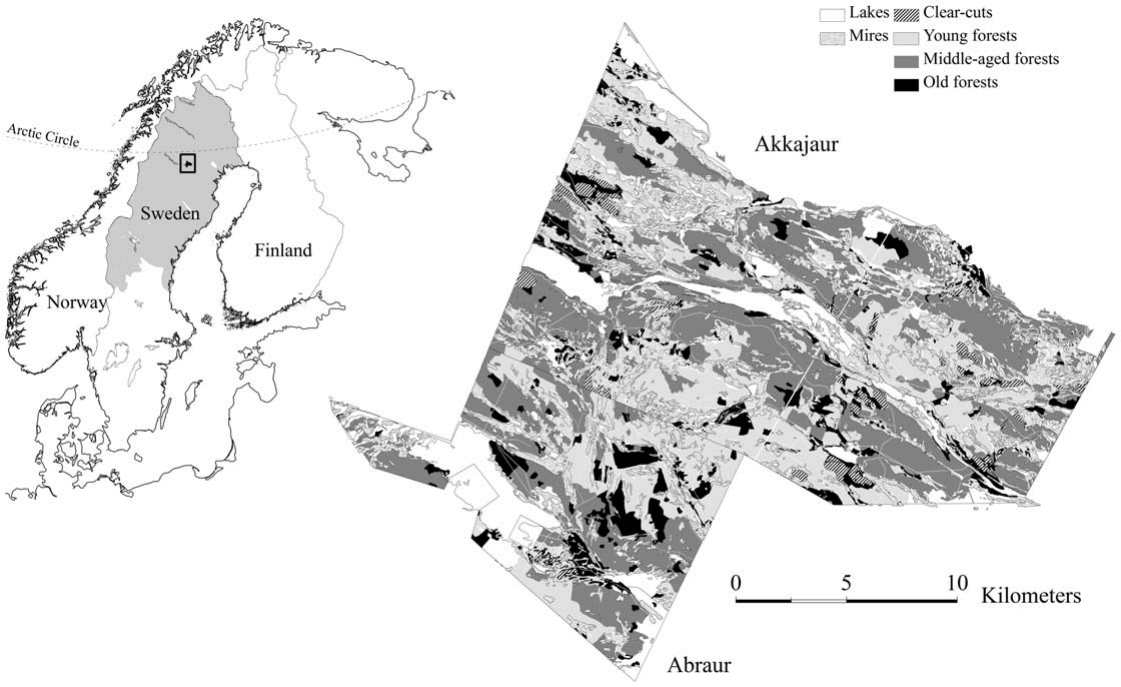
Location of the study area. The gray area in the overview map illustrates the Swedish reindeer herding area.

Located at latitude 66°20′N, mid-boreal vegetation characterizes the study area of ca. 26,600 ha (Fig. 1). Its forests are dominated by Scots pine (*Pinus sylvestris*), with a few stands dominated by Norway spruce (*Picea abies*). Deciduous trees, such as birch (*Betula pendula*), goat willow (*Salix caprea*), and aspen (*Populus tremula*), are present but not common. The ground layer consists of dwarf shrubs (*Juniperus communis, Vaccinum myrtillus, Vaccinum uliginosum, Empetrum nigrum*), mosses, and ground lichens (*Cladonia* spp., *Cetraria* spp.) growing on dry glacio-fluvial soils. Several small lakes and mires are scattered across the landscape, covering ca. 24% of the area. The river Pite Älven runs through the area, and was formerly an important means of transporting timber. The site's elevation varies from 277 m above sea level along the river valleys to 550 m at moraine hilltops. The study area, which consists of former National Forests Akkajaur and Abraur (hereafter referred to collectively as Akkajaur), is used for year-round reindeer grazing by the Sami herding districts Östra Kikkejaur and Ståkke, and as a migration corridor and winter grazing area by the Luokta-Mavas herding district. In 1936, Akkajaur was extended northwards, increasing its area by ca. 17% (4,620 ha) to its current size.

### Data collection

Araújo & Williams [30] identified local-intrinsic factors, such as suitable habitat, and regional-intrinsic factors, such as colonization from other areas based on the connectivity of similar habitats within the landscape, as essential for the persistence of a species. In this study, we focus on local-intrinsic aspects, by which we mean the characteristics of the forest at the stand level.

Field work was conducted in August 2009 and 2010. Using digitized maps of the study area in a geographical information system (GIS), we stratified forest stands into 9 classes according to their age (young = 1–39 yrs, mature = 40-120 yrs, and old = >120 yrs) and species composition (pine, spruce, mixed) on the basis of inventories conducted by the forest's owner, Sveaskog (Table 1, see also 11). A stand was denoted as a pine or spruce stand if the species in question accounted for 80% or more of the stems.

**Table 1:** Number of sampled forest stands in strata, separated by dominant tree species and age (total forest stands N = 852, sampled forest stands n = 144).

	Pine			Spruce			Mixed		
	Stratum total	sampled	% of sampled	Stratum total	sampled	% of sampled	Stratum total	sampled	% of sampled
**Young**	177	24	17	2	1	1	35	10	7
**Mature**	352	48	33	1	1	1	44	12	8
**Old**	177	28	19	13	7	5	51	13	9

The sampling units were positioned between 500 m and 20 m away from the nearest forest road so as to avoid road effects. Within this buffer zone, forest stands were selected by random sampling. Circular sampling plots, covering ca 0.1 ha, were semi-randomly distributed in the selected forest stands of the 9 strata (Table 1). Plots were located in the field with a GPS receiver. At that point, the basal area of the respective forest stand was measured using a relascope, and the height of a representative tree in the stand was recorded.

The canopy closure at each sampling plot was calculated from a single digital hemispheric photograph taken at each plot with a fish eye lens, after conversion to binary images using the program Gap Light Analyzer [31]. As *Bryoria fuscescens* is preferred forage by reindeer [20], we focused on this species. *Alectoria sarmentosa*, which was also present in the area in low abundance, prefers later successional spruce and moister habitat stands than the dry pine forests of Akkajaur [32], [33]. The biomass of *Bryoria fuscescens* in the stand was estimated visually and classified into one of 4 abundance categories, representing mean values of arboreal lichen biomass: none (0), sparse (1; ≤35 kg ha^−1^), moderate (2; 35–120 kg ha^−1^), and abundant (3; ≥120 kg ha^−1^). This classification system was based on the findings of Helle *et al.* [34], who used an oven-dried lichen clump of known mass to classify lichen biomass using these four categories. As the clump method underestimates biomass of arboreal lichens in trees, the bias introduced by visual estimation has to be corrected. In the method of Helle *et al.* [35], this was done by using regression equations, that compare the estimated biomass on a branch to the oven-dried biomass removed from the same branch.

Stands containing no lichens were assigned to category 0. Category 1 was assigned to stands in which individual lichen thalli were detected on a small number of trees and in low quantities. Stands in which lichen clumps were readily apparent on each tree were assigned to category 2; none of the studied stands contained enough lichens to be assigned to category 3. Lichen abundance was thus averaged over the trees within the sampling plot, and this average was taken as the stand level. Using the same method, Jaakkola *et al.* [35], found a maximum lichen biomass on whole trees of 474 kg ha^−1^ at fresh sites in mature spruce forests.

In order to totally eliminate edge effects, e.g. from neighboring open areas, it would have been necessary to focus exclusively on sampling plots with a distance of 50 m in every direction to neighboring stands [33], which would require a minimum area of 0.79 ha assuming a circular forest stand. However, the forest patches in the study area were not always shaped in a way that would make this possible, especially in the older forest stands. However, sampling plots in close proximity to open places such as larger lakes or mires were not common in our dataset.

In total, we sampled 144 forest stands. One third of the sample plots were in mature pine forests, 19% were in old pine forests, and 17% were in young pine stands. The remaining plots were located in less abundant forest types (Table 1). Stands dominated by spruce were especially rare.

It has previously been reported that there is a strong relation between forest age and arboreal lichen biomass [33], although other authors have suggested that the correlation with volume is stronger [35]. Stand age was strongly correlated with many other forest characteristics specified in the forest owner's stand registry (Table 2) and with the canopy closure calculated from our fish –eye photo analysis. We chose to use age as a predictor variable in our models, since it can easily be compared across different studies and is recorded in most forest databases.

**Table 2:** Two-tailed Spearman's rank correlations (r_s_) between sample stand characteristics, n = number of sampled stands.

	Forest Age (yrs)	Volume (m^3^/ha)	Basal Area (m^2^)	Diameter (cm)	Height (m)	Canopy Closure (%)
**Forest Age**	1	-	-	-	-	-
**n**	144					
**Volume**	0,776**	1	-	-	-	-
**n**	144	144				
**Basal Area**	0,512**	0,898**	1	-	-	-
**n**	134	134	134			
**Diameter**	0,898**	0,712**	0,435**	1	-	-
**n**	125	125	125	125		
**Height**	0,856**	0,904**	0,654**	0,878**	1	-
**n**	144	144	134	125		
**Canopy Closure**	0,561**	0,611**	0,526**	0,304**	0,556**	1
**n**	144	144	134	125	144	144

**Correlations are significant at the 0.01 level.

Permission for conducting the study was given by the forest owner Sveaskog. No endangered or protected species were involved in the study.

### Statistical analysis

We used binary logistic regression analysis to model the abundance of lichens in relation to forest stand age. This was done by converting the arboreal lichen abundance classes into dichotomous data. Two different approaches were adopted: comparing plots in which no lichens were present (i.e. those in category 0) to those with at a lichen class of at least 1, and comparisons between plots with a lichen class of 1 and those with a lichen class of 2.

In a logistic regression analysis, the dependent dichotomous variable *x* (i.e. the presence [1] or absence [0] of lichens) is not modeled directly. Instead, the regression estimates the probability *π(x)* that the outcome will be “positive”, i.e. that lichens will be present in a particular plot for a given value of the independent variable. A logistic regression takes the form:
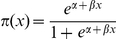
Both the constant *α* and the predictor coefficient *β* are fitted by the logistic model; the coefficient *β* gives the slope of the regression. The output of the model ranges from 0 to 1 for each stand, with an increase in the probability, *π*, corresponding to a positive outcome.

The model's coefficients are most easily interpreted in terms of odds ratios (OR). An OR measures how likely an event (such as the presence of lichens) is relative to its opposite (in this case, the absence of lichens) for any given value of the independent variable. The OR thus indicates the rate of change in *π(x)* for a one unit change in the independent variable *x* [36], [37]. An OR larger than 1 indicates a positive relationship between the dependent and independent variables; values below 1 indicate the opposite. The odds ratio is obtained as follows:
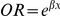



### Model fit, cut-off selection and model validation

We investigated the models' goodness-of-fit, i.e. the accuracy of their classification of occupied and unoccupied sites, using a Receiver Operating Characteristic curve (ROC). This curve is constructed using the fraction of correctly classified events, i.e. the true positive fraction (sensitivity) and true negative fraction (specificity) for every threshold value that could potentially be used to discriminate between occupied and unoccupied sites. Plotting the sensitivity against (1 – specificity) for all possible values calculated by the model, a ROC curve is derived [38], [39]. This curve illustrates the model's ability to discriminate between occupied and unoccupied sites over the whole range of possible threshold values that could be used to segregate the data set. If the model is unable to discriminate between the site types, the ROC - curve will be a straight line with a gradient of 45° and the model outcomes are no more reliable than chance. The closer the curve is to the left and top axes, the better its discriminative ability and the better the model fit [40]. Pearce & Ferrier [38] suggest that the area under the ROC curve (AUC) is an indicator for the model's discriminative ability; an AUC between 0.5 and 0.7 indicates weak discriminative ability, while a value between 0.7 and 0.9 indicates reasonable discrimination and values exceeding 0.9 indicate a very good ability to discriminate between occupied and unoccupied patches using the binary classification scheme.

The model values calculated using binary logistic regression, represent continuous probabilities of occurrence. To classify a forest type as being occupied or unoccupied by lichens, the model values have to be split at a certain threshold value. The ROC curve illustrates the compromises that are made between the true and false positives as the decision threshold is changed, making it possible to identify an optimal threshold using the sensitivity and specificity [38]. Our strategy for finding the most robust cut-off point was to split the data set at the point where the sum of the sensitivity (true positives) and the specificity (true negatives) is at its maximum, i.e. the value that gives the highest rates of correct classification of occupied and unoccupied sites.

The model's performance was tested using cross-validation [39], [41]. The data set was randomly divided into two parts - a training set consisting of 80% of the data and a test set consisting of the remaining 20%. To assess the consistency between the predictions of the training model and the test model regarding the presence of lichens, the corresponding model outcomes were binned into 10 equal-sized classes (0-0.1; 0.1-0.2;…; 0.9-1). A stable model would be expected to have a similar proportion of its total outcomes in each bin for both the training and the test set. Significant Spearman's Rank correlations (*r_s_*) between the frequencies for the two sets indicate that the model is applicable to both the training and the test set [39]. We repeated the procedure of random data-partitioning and correlation of bin-frequencies four times, and calculated the average correlation coefficient and significance of the five resulting correlations.

### Compilation of historical forest age data

Historical forests inventories for the years 1926, 1936, 1960 were obtained from archival sources [11], and the most recent inventory, for 2006, was obtained from the forest company Sveaskog. These inventories differ in both the quality and quantity of the reported forest characteristics. For instance, the way in which forest age is calculated differs, as the scope of forestry shifted from selective cutting to clear-cutting in the 1950s [11]. While the mean age of forest stands is available for the most recent survey year (2006), older data from 1926, 1936 and 1960 had to be interpreted in different ways. The 1926 and 1936 inventories recorded the age for each forest stand as a composite of 5 age classes, each covering a 40-year interval (1-40 years, 41-80 years, …, 120-160, >161 years). These sources did not provide any granularity with respect to the ages of stands older than 161 years because forests of that age were left unmanaged. The older sources provide data on the percentage of the area of each stand covered by trees in each age bracket. This flexible classification scheme reflects the variable age structure typical for forest patches at that time. Using these percentages and the mean of each 40 year - age class, an area-weighted average age was calculated for each forest stand. The hypothetical maximum age for a stand that could be calculated using this scheme would be 161 years, for a stand that was entirely covered by trees within the highest age bracket. Therefore, all age estimations were constrained by this upper limit, even those derived from more recent inventories in which older mean ages were reported. The forest age data for the 1960 inventory are reported using a classification scheme consisting of ten age classes at the stand level, each of which spans 20 years. This reflects a change in forest management practices that favored more evenly-aged stands [24]. Due to these restrictions, forest ages derived from the historical inventories should be regarded as approximations of the real situation in those times.

### Simulation of future management scenarios

We chose three possible scenarios for future forest management, all differing in their impact on forest age structure and thus on potential arboreal lichen abundance. The first was a “business as usual” scenario (BAU), in which it is assumed that the forests will be managed as they currently are by their owners, with the intent of satisfying contemporary timber management and nature conservation objectives. The second involves more intensive forestry (INT), while the third involves a total absence of management, i.e. no forestry whatsoever (NO). All three scenarios were developed using the StandWise software package, which is part of the Heureka system. This system was developed by the Swedish University of Agricultural Sciences (SLU) for forest management planning and analysis on several scales [42], [43]. For BAU and INT the economic outcome of forestry was maximized in terms of the Net Present Value (NPV), i.e. the sum of all discounted future net costs and incomes (for a real interest rate of 2,5%). An even flow of timber harvests was ensured by penalizing fluctuations in harvests in the optimization problem goal function. The best management alternative among the set of potential management alternatives for each stand was found by solving the optimization model using linear programming. The behavior displayed under the three different scenarios was subjected to the age distribution of forest stands given in 2006. The following assumptions were made for the different scenarios:

- In BAU, areas currently under protection were left unmanaged, as were forests older than 160 yrs (total covered area: 2268 ha). For stands older than 120 years, rotation periods were prolonged and they were not allowed to be final felled until after the next ten year period (total covered area: 1848 ha); complete final felling throughout this second area immediately after the end of these ten years was prevented by the even timber flow criterion in the goal function. Other stands were assumed to be managed according to their current management regimes in terms of thinning programs and final felling, including 1-2 thinning events and a final felling age of around 100 years. The minimum final felling age was that stipulated by the Forestry Act, i.e. typically 90 years.
- INT assumes that all stands can be cut, including those in protected areas or that are more than 160 years old. Stands older than 120 years were allowed to be cut immediately, which resulted in shorter initial rotations compared to the BAU scenario.
- NO is a simple extrapolation of forest aging into the future, without considering any disturbances of patch dynamics, whether human or natural in origin. In reality, unmanaged forests are subject to natural disrupting events such as forest fires or insect breakouts, which mean the forests are maintained in a dynamic state with diverse succession phases.

### Assessment of landscape fragmentation

To analyze the degree of fragmentation, we imposed a buffer of 200 m around forest stands identified as threshold patches by the model (see above). This buffer was based on the potential for dispersal of arboreal lichens as reported by Dettki & Esseen [44]. In other sources, the dispersal distance has been reported to range from 100 m, with the number of dispersed thalli decreasing by 50% from old growth forests [45], to 350 m from forest stands with high lichen abundance [46]. We also calculated the largest patch size index (LPI) in Fragstats [47] to quantify the change in the area coverage of connected threshold stands identified by the regression model.

The general fragmentation of all forest stands present in Akkajaur due to forestry was calculated as the area-weighted average stand size (*S_a_*). This is an appropriate method of averaging when the frequency distribution of large and small stands is skewed [48]. Using *A_i_* to refer to the size of the *i*-th stand, it is calculated as
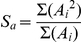
Because we compared changes in the age distribution within the forest over several decades, it was necessary to ensure that the data for different years was comparable. Administrative decisions and the effects of forestry have caused the number of forest stands, their spatial configuration, and the size of the study area to change over the studied period of time. Therefore, changes in the size and quantity of forest stands of a given age have to be considered to properly understand the forestry-driven landscape dynamics of the area and their influence on the behavior of the logistic regression model. For instance, large stands of young trees contribute more to the landscape configuration than many small stands of much older trees, but the latter would have a more pronounced influence on the regression model because of their age. To account for these temporal changes in the spatial configuration of Akkajaur in terms of the proportion of stands of different ages and their influence on the model, we calculated an “area-weighted model”. In this model, the raw modeled value for a given stand, being only depended on stand age, is multiplied by its relative size as a percentage of the total study area. This scales the purely age-dependent model value for a given forest stand according to the magnitude of its spatial contribution to the landscape pattern. Although dimensionless, the area-weighting gives a better understanding of how the landscape may be “perceived” as an arboreal lichen habitat. All statistics were computed using PASW Statistics (version 18).

## Results

### Modeling arboreal lichen abundance in forest stands

Nearly half of the sampled stands were without arboreal lichens (45%). Low arboreal lichen abundance (abundance class 1) was recorded in 34% of all stands, while arboreal lichens were moderately abundant in the remaining 21% (abundance class 2; Table 3).

**Table 3:** Lichen classes and their subdivision in abundance classes as recorded at sampling plots in forest stands, Abundance Classes 1 and 2 both belong to Lichen class “Present”.

Lichen Class	Abundance Class	Mean Biomass (kg/ha)	N	%
**Absent**	0	0	65	45
**Present**	1	35	49	34
	2	120	30	21

**Figure 2:**
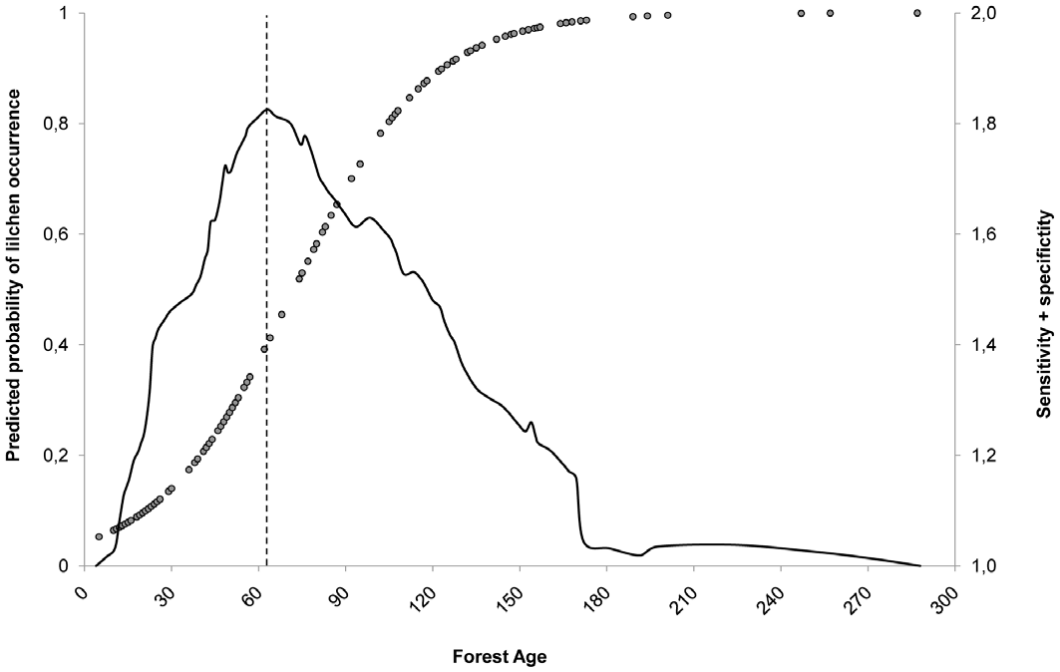
Predicted probability of arboreal lichen presence and cut-off point selection. Results from the logistic regression in circles, sensitivity plus specificity as solid line and the chosen forest age cut-off for discriminating between stands with and without lichen as dashed line.

Binary logistic regression analysis successfully discriminated between stands that did and did not contain lichens (i.e. between those in class 0 and those in either class 1 or class 2), using forest age as the independent variable (Table 4). Exponentiating the regression coefficient for forest age gave an odds ratio of 1.04. This indicates that the likelihood of the stand containing lichens increases by 4% per year of age. Fig. 2 illustrates the increase in the predicted probability of lichen occurrence (*π*) with forest age. No model capable of achieving statistically-significant discrimination on the basis of age between stands in class 1 and class 2 could be identified; all of the models examined exhibited low AUC values (0.64), indicating poor discriminatory ability (data not shown). None of the other forest variables (Table 2) produced significant models either, probably due to the low sample size of forests in which *Bryoria* was abundant (N = 30, Table 3). All of the remaining analysis and discussion therefore focuses on the presence-absence model.

**Table 4:** Regression coefficients for predicting the presence of lichens on the basis of forest age.

	Coefficients (β)	S.E.	df	Sig.	Nagelkerke R^2^	Goodness of fit χ2	Sig.	Exp(β)	95% C.I.for Exp(β)	
									Lower	Upper
**Forest Age**	0,04	<0,01	1	<0,001	0,63	20,116	<0,001	1,04	1,03	1,06
**Constant**	-3,11	0,53	1	<0,001				0,045		

### Model fit, threshold value selection, and validation

The large area under the ROC curve (AUC: 0.92; *p*<0.001, SE = 0.03) demonstrates the model to be well-fit; it correctly discriminates between stands with and without lichens on the basis of stand age in 92% of the cases examined. The sum of the sensitivity (true positives) and specificity (true negatives) peaked at a forest age of 63 years, which was used as the cut-off point (Fig. 2). The replicated cross-validation of the logistic model resulted in a highly significant Spearman-correlation (*p*<0.001; correlation coefficient *r_s_* = 0.945) between the test and training sets, confirming the model's robustness. The ability of the model to estimate the historical probability of lichen abundance is limited by the fact that the historical sources used did not record the ages of trees older than 160 years, which introduces some bias into the model. However, the probability that arboreal lichens will be present in stands of that age is 0.97, and so the artificial limit imposed by the older data has only a small effect on the estimated probability that lichens were present in historical stands.

### Age threshold

Our model identified the stand age threshold beyond which lichens are more likely to be present than absent as 63 years (Fig. 2). This cut-off point is also reasonable to apply in light of the historical data, since all of the different age classification schemes used in the historical inventories included an age class for trees “older than 60”. We therefore used a cutoff of 60 years in the subsequent analyses, to retain consistency with the historical data.

**Figure 3:**
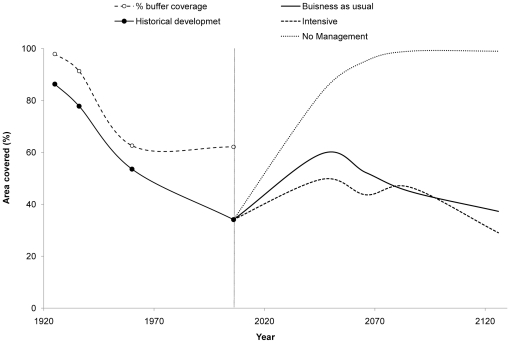
Past and potential future changes in the area covered by stands older than 60 years. The increasing fragmentation from 1920 to 2006 is illustrated by increasing area isolating stands older than 60 years from each other, expressed as area covered by a 200 m-buffer.

Over time, the proportion of forests older than 60 years decreased, from 86% cover in 1926 to 34% in 2006 (Fig. 3). Consequently, there is little connectivity of these forest patches, which restricted the scope for lichen dispersal, especially after 1960. As these older forests became less common and ceased to occur in close proximity to one-another, the buffer zone of 200 m around the forest patches increased relative to the area covered by these older stands from 1960 onwards (Fig. 3). The largest connected forest patch covered 50% of Akkajaur in 1926, but only 6% in 2006 (Table 5). The 74% decrease in area-weighted mean stand size S_a_ underlines the increasing isolation of these above-threshold stands from their nearest similarly-aged neighbors (Table 5).

**Table 5:** Largest patch index (LPI) in % and area-weighted mean stand area (ha) for above-threshold stands.

	1926	1936	1960	2006
**LPI (%)**	50.2	39.8	24.3	5.9
**S_a_ (ha)**	163	155	160	43

Generally, variation in stand number and size is a measure of fragmentation, since the creation of more discrete stands in the same area splits the area into smaller parts. After the extension of Akkajaur in 1936, the overall stand number rose by 12% in 1960, and by another 28% in 2006 (Table 6). As a result, the mean stand area decreased by 40% between 1936 and 2006. The area covered by stands older than 140 years decreased by 50% between 1926 and 2006.

**Table 6:** Changes over time in the stand number (N), mean area (ha), standard deviation and total area (ha).

	1926	1936	1960	2006
**N**	696	575	651	900
**Mean area (ha)**	32	49	44	30
**Mean area Std. Dev**	62	77	64	30
**Total area (ha)**	21,979	28,283	28,869	26,600

### Future scenarios

We used the 60-year threshold to quantify the consequences of three different future approaches to forest management, one corresponding to intensive forestry, one to “business as usual”, and one in which no management was undertaken. Fig. 3 illustrates the incidence of forest stands older than 60 years in the three scenarios.

Under the “business as usual” scenario, the area covered by stands that provide a suitable habitat for lichens will increase by about 30% relative to the present day after 40 years, as the many young stands mature. However, future harvesting will reduce this level back to that which currently obtains after a period of 120 years. More intensive forestry is projected to reduce the coverage of lichen stands, as logging would be focused on the older, previously-protected forest stands. This would only reduce the size of the lichen habitat by 5% relative to the present day, but would generally result in a smaller lichen habitat than the BAU scenario over time. Conversely, leaving the area unmanaged would increase the area of the lichen stands to almost their former extent within 40 years. However, it should be noted that this scenario does not account for natural dynamics such as forest fires and gap dynamics, which would also affect lichen dispersal, establishment and abundance.

### Changes in age distribution and forest patterns

**Figure 4:**
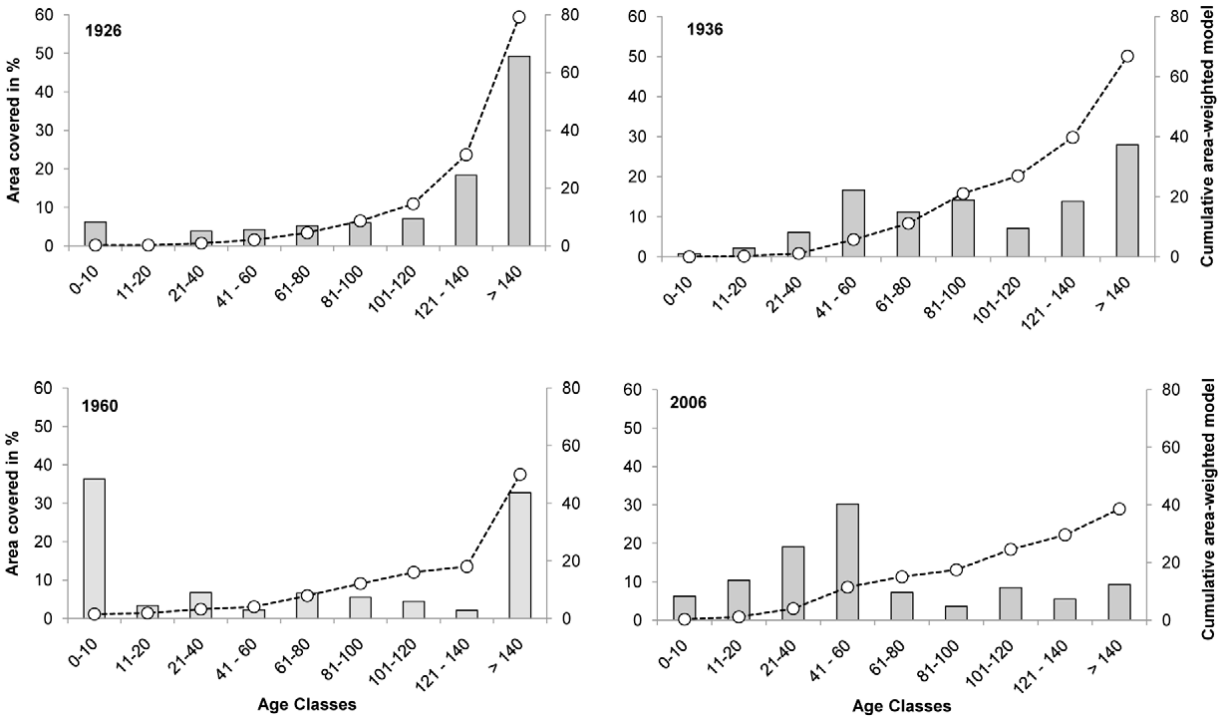
Changes in age composition in Akkajaur 1926–2006. Area in % covered by age classes illustrated by columns, their cumulative contribution to the area-weighted model by the dashed line.

In 1926, Akkajaur was dominated by older stands (>140 yrs), which covered nearly half its total area (Fig. 4). This proportion had decreased to only 9% in 2006, when the landscape was dominated by stands younger than 60 years (Fig. 4). Between 1926 and 1936, selective logging of older trees reduced mean stand age (Fig. 4). Extensive clear-cutting between 1936 and 1960 reduced the area covered by all age classes, giving rise to a bimodal distribution of clear cuts/young stands together with old stands (Fig. 4).

### Area-weighted model: accounting for landscape changes

The model's output values are directly dependent on stand age. However, calculations based on stand age alone cannot properly represent the capacity of the whole landscape to sustain arboreal lichens, because they do not account for the size of individual stands relative to the total area. Consequently, the area-weighted model was used to compare the historical trends in the model values due to forestry in a way that reflects both stand age and spatial area. Fig. 4 shows the cumulative distribution of results calculated using the area-weighted model, i.e. the contribution of each forest age class to the sum of the area-weighted model values for each year studied.

In the years prior to 2006, forests older than 120 years contributed substantially to the area-weighted probability of lichen occurrence, *π*. Landscape fragmentation, i.e. the decreases in the number and stand size of older forests and the decreasing stand ages towards 2006 had a negative effect on π at the landscape scale. The cumulative curves become less steep from year to year, i.e. the influence of older age classes on lichen occurrence (*π*) decreases due to the reduction in the area of land they cover. The cumulative area-weighted model indicates that their influence decreased by 51% between 1926 and 2006, because forest stands with a high probability of lichen occurrence became both smaller and less abundant (Fig. 4). In contrast to the situation in 1960, the high abundance of forests aged between 40 and 60 years in 2006 could not compensate for the loss of very old forests.

## Discussion

### The cut-off point and its practical relevance

The historical land usage patterns of the Sami people in extracting subsistence resources have shaped the boreal forests of Sapmi into a “cultural landscape” *sensu* Berkes & Davidson-Hunt [49]; such uses include the removal of the inner bark from pine trees to act as a plant food resource [50], [51] or cutting lichen-rich trees as emergency food for reindeer in harsh winters or for other herding practices [52]. In contrast, commercial timber extraction turned the forests into an “economic landscape” that in which the availability of arboreal lichens that sustain reindeer herding is limited compared to the conditions prior to the intensification of forestry. Our analysis has quantified some of the impacts forestry has had on arboreal lichens in Akkajaur.

Rather than modeling *Bryoria* biomass in relation to stand age [33], [44], we modeled the probability of lichen occurrence [53]. This identified the value and importance that an area might possess, making our approach analogous to that adopted by Juutinen *et al.* [54] in their evaluation of the conservation value of a given forest. A threshold age of 63 years was identified for our study area, with its particular history of timber harvesting; under current conditions, *Bryoria* is very likely to be present in stands above this age (Fig. 2). This threshold could conceivably be used as an indicator to evaluate the potential for sustainable reindeer husbandry.

Similar threshold ages were reported by Goward & Campbell [55] for an unmanaged stand of *Abies lasiocarpa*, *Picea engelmanni* and *Pinus contorta* in east-central British Columbia. In these trees, it took 60 years for *Bryoria* to grow as far as the outer tips of the tree branches, which is the point when lichen accumulation begins. Likewise, Stone *et al.* [56] found a significant increase in lichen biomass 70 years after harvesting in eastern Canada. In a spruce and pine dominated forest in south-central Sweden, Uliczka & Angelstam [57] recorded that *Bryoria* fragments were present on 5% of sampled trees aged between 61 and 80 in circular plots of 10 m radius (N = 90).

This threshold needs to be tested in other parts of the Fennoscandian reindeer herding area before it can be generally applied. For instance, sites with higher productivity are dominated by spruce [22], leading to niche partitioning between *Alectoria sarmentosa*, which prefers the lower canopy, and *Bryoria fuscescens* in the upper canopy [58]; this changes the growing conditions. We therefore recommend that our model should be tested in a diverse set of habitat types to identify additional habitat-related variables that influence the occurrence of arboreal lichens.

As our results show, maintaining both the spatial and temporal coverage of a key habitat, i.e. forest older than 63 years, can increase the likelihood that lichens will be present as a grazing resource for reindeer. Because the probability of lichen occurrence increases with age, extending rotation times could greatly increase the abundance of arboreal lichens [27], [44]. Further, the continuity of key forest habitats is central to the maintenance of high lichen abundances: Esseen *et al.* [33] showed lichen biomass to be higher in un-harvested old growth forests than in selectively logged forests of the same age Therefore, temporal and spatial continuity is especially important; the availability of suitable forest stands alone does not guarantee the presence of *Bryoria*, especially when opportunities for colonization have been limited during the forest's history [59].

### Forest history: Loss of important landscape elements

On the landscape scale, forest stands in Akkajaur were consecutively fragmented into ever-smaller stands over the 80 years examined in this study. This affected old forest stands in particular (Fig. 4), and the mean stand age decreased substantially (Table 6). As a result, the probability of arboreal lichens being present in a given region decreased due to losses of valuable habitat and reduced connectivity between above-threshold stands in space and time. In Akkajaur, the post-1960 loss of the remaining large continuous old forests, which had a high probability of lichen occurrence constitutes a “bottleneck” in habitat availability and reduced the landscape's ability to provide arboreal lichens as forage for reindeer. The contribution of old forest stands to the probability of arboreal lichen being present at the landscape level in 2006 is therefore low in terms of area (Fig. 4).

Since *Bryoria* is a species with limited dispersal abilities, the spatial structure of its habitat is essential for its survival and ability to colonize new patches [45], [60]. Because stochasticity in its survival and reproduction may affect population sizes [61], changes in its spatial and temporal habitat patterns are especially important for understanding the impacts of fragmentation and habitat loss. In particular, small and/or isolated patches do not readily recover from environmental disturbances, nor do they facilitate immigration and establishment. This is the situation in many boreal forests in both Scandinavia and Canada [62]. The reduced viability of the lichen population may therefore result in local extinction due to stochastic events, e.g. when patches are too isolated to be colonized [61]. Further, a species' response to a specific disturbance may be subject to time lag. This makes it particularly important to study historical landscape patterns, as they can explain modern day species distribution patterns [63]. Historical landscape transformations therefore create a path dependence, i.e. future options and changes depend on decisions taken both in the past and at present [64]. Because of the slow dynamics of forests, management decisions will impact their spatial and temporal development on long (multi-decade) timescales, and will limit or facilitate future options (Fig. 3). Our future projections illustrate the possible outcomes of some different management approaches.

### Management scenarios

The projected effects of the three different forestry management scenarios on the abundance of threshold stands provides insight into the likely future development of the forests in question, which are particularly important habitats for arboreal lichens (Fig. 3). Under both the business-as-usual and the intensive scenarios, the coverage of threshold stands will initially increase relative to the present day situation, because of the current predominance of young and middle-aged stands today. The BAU approach is focused on maintaining a constant timber harvest. However, it has different consequences for arboreal lichens. Under BAU, forests older than 160 years are left unmanaged, as are protected areas. These areas will therefore serve as “source habitats” for lichen dispersal. Due to the dynamic pattern of emerging and disappearing threshold stands, lichen establishment and persistence will be mainly restricted by the temporal and spatial availability of colonizing habitats.

Under the intensive scenario, all of the areas that are left unmanaged in the BAU scenario are available for harvesting. This results in a loss of lichen-rich stands and is also likely to further decrease the dispersal ability of the lichens due to more pronounced fragmentation. As such, although some forests above the threshold age are retained under this scenario, they are less likely to be colonized to the same extent as would be the case under BAU. One could reasonably speculate that the age threshold might shift upwards, although such older trees would be more likely to be harvested. It should be noted that this management strategy is not allowed under the current Swedish Forestry Act.

The continuity of forest cover increases under the No Management scenario. This strategy would support the spread of arboreal lichen fragments or dispersal units and consequently promote their establishment. At the same time, lichen abundance would increase in already-colonized habitats. This, however, does not mean that potential lichen occurrence under this scenario is similar to the situation in 1926. On the landscape scale, the forests will still be considerably younger and may not have had enough time to accumulate lichen biomasses as high as those that would probably have been common in 1926.

The age threshold of 63 years was derived from the landscape and stand patterns seen in Akkajaur today. Over the 20^th^ century, forests have changed in terms of their age distribution and in their standing volume, which almost doubled in middle-aged second-growth forests compared to that at the beginning of the 20^th^ century [11]. Being denser and thus darker, these second-growth forests give rise to different growing conditions for arboreal lichens in terms of parameters such as the availability of light and water [60]. Therefore, the threshold of 63 years, which was calculated using present-day data, might result in underestimation of the historical abundance of lichen in the years before clear-cutting became the dominant practice and gave rise to denser forests.

### Management implications

As our management scenarios show, current management will inevitably further decrease the potential occurrence of arboreal forage for reindeer in the long term. To create arboreal lichen habitats for the future, it will be necessary to manage these forests carefully without sacrificing their economic viability.

Arboreal lichens require substrates to grow on and are thus dependent on the structure of their host trees. In an east-Canadian black spruce forest, the number of branches decreased in very old trees (>200 yrs), leading to reduced lichen biomass compared to intermediate succession stages (101–200 yrs) [65]. Structurally-diverse forest stands with trees of varying age classes would therefore be expected to be most suitable for the establishment and persistence of lichen in the landscape [66], [67].

The growing conditions for arboreal lichens and habitat diversity on the stand level can be improved by reducing the differences in structural heterogeneity between natural and managed stands. Because forestry focuses mainly on the forest stand level, management practices do not consider the larger landscape level at which reindeer husbandry operates to meet the reindeer's season-depended habitat requirements [15]. At the landscape scale, managed forests with relatively short rotation times are in some aspects more diverse than natural forests, particularly due to pronounced edge effects between diverse age classes [27]. Increasing the amount of older forests provides habitats suitable for colonization and maintains trees harboring lichens that can serve as dispersal sources [68]. Although both forests and lichens grow slowly, the rapid dynamics of industrial forestry, which are reflected in short rotation times among other things [8], have overtaken the slower dynamics of lichen ecology that are fundamental to reindeer husbandry. Extending rotation periods beyond the currently used 100-120 years, and their variation on the landscape scale, will be necessary to allow sufficient biomass accumulation to provide forage for reindeer [69], [44].

### Conclusions

The two different users' divergent perceptions of the forest ecosystem and its resources – timber and lichens – have given rise to a mismatch in the temporal and spatial dynamics of those resources, which has rendered the current landscape pattern less favorable to arboreal lichens than it has been in the past. Our results show that management decisions taken at a given point in time will affect the quality and quantity of arboreal lichen habitats for long periods of time, and will thus have similarly long-lasting effects on the sustainability of reindeer herding. Emphasis should therefore be placed on the need to consider ecological and economic values (e.g. for timber) as well as sociopolitical factors, such as indigenous interests, when making forest management decisions that affect the allocation of resources between different interests groups [70], [71]. Restoring the availability of arboreal lichens as a reliable grazing resource is dependent on forestry management decisions at the stand and landscape scale. Because we found 63 years to be the minimum age at which forest start accumulating lichen biomass, future studies should focus on giving guidelines to managers how to incorporate this age threshold into forestry to establish necessary habitat for arboreal lichens. This could be done e.g. by simulating prolonged rotation times, aggregation of old stands to reduce negative edge effects and fragmentation and analyzing economical consequences of changed timber harvest resulting from such modifications.
